# Porous Layered Double Hydroxides Synthesized using Oxygen Generated by Decomposition of Hydrogen Peroxide

**DOI:** 10.1038/s41598-017-00283-9

**Published:** 2017-03-28

**Authors:** P. Gonzalez Rodriguez, M. de Ruiter, T. Wijnands, J. E. ten Elshof

**Affiliations:** 1 Materials innovation institute (M2i), Elektronicaweg 25, 2628 XG Delft, The Netherlands; 2 Inorganic Materials Science Group, MESA+Institute for Nanotechnology, University of Twente, P.O. Box 217, 7500 AE Enschede, The Netherlands

## Abstract

Porous magnesium-aluminium layered double hydroxides (LDH) were prepared through intercalation and decomposition of hydrogen peroxide (H_2_O_2_). This process generates oxygen gas nano-bubbles that pierce holes in the layered structure of the material by local pressure build-up. The decomposition of the peroxide can be triggered by microwave radiation or chemically by reaction with iodide (I^−^) ions. The carbonate LDH version [Mg_0.80_Al_0.20_(OH)_2_](CO_3_)_0.1_∙mH_2_O was synthesized by microwave-assisted urea coprecipitation and further modified by iodide or H_2_O_2_ intercalation. High resolution Scanning Electron Microscopy (HR-SEM) and Brunauer-Emmet-Teller (BET) analysis were used to assess the morphology and surface area of the new porous materials. The presence of H_2_O_2_ in the interlayer region and later decomposition triggered by microwave radiation generated more pores on the surface of the LDH platelets, increasing their specific surface area from initially 9 m^2^/g to a maximum of 67 m^2^/g. X-Ray Diffraction showed that the formation of the pores did not affect the remaining crystal structure, allowing possible further functionalization of the material.

## Introduction

Extensive research has been dedicated in recent years to improve the architecture of micro and mesoporous structures for applications in multiple fields. Particularly, layered ceramics and their pillared derivatives have shown promising qualities as catalysts^1, 2^, ion exchangers^3, 4^ and gas absorption systems^5^. The relevance of these materials is related to their broad range of compositions, versatile synthesis method and high potential of functionalization^6^. They typically consist of crystals with a platelet shape that expose their outer surfaces to the medium. Sometimes these crystals are highly aggregated, exhibiting relatively low surface areas, which requires a modification in order to increase their active surface area. This can be done by the intercalation of bulky molecules that give rise to pillars^7^, or the addition of active entities that participate in the final application. Polyoxometalates such as Keggin-type ions [AlO_4_Al_12_(OH)_24_(H_2_O)_12_]^7+^ (Al_13_) and metal alkoxides such as tetraethyl ortosilicate (TEOS) have been incorporated by ion-exchange/intercalation into various layered structures such as Layered Double Hydroxides (LDH)^8, 9^ and manganese oxide (MnO_2_)^10, 11^. The intercalation is usually followed by thermal treatment in order to form pillars from the intercalated species, rendering microporous openings in the layered structure^12^.

The surface area of these layered systems can also be increased by exfoliation. Surfactants reduce the interaction between the layers and may be used to obtain dispersions of thin nanosheets^13^. Nevertheless, when the powders are dried, the exfoliated dispersions collapse and the layers re-stack, not necessarily giving rise to a substantial increase in surface area in comparison with the starting situation^14^. These processes sometimes require toxic solvents and multiple process steps^15, 16^. Moreover, exfoliated systems cannot always retain the functionality of the intercalated compounds because the additives are susceptible to removal by external agents, e.g. solvents and photodegradation.

In this work we propose a method to increase the porosity of LDH compounds without pillarizing the interlayer region or use of toxic solvents, while avoiding the presence of residual chemical waste. The method is based on hydrogen peroxide (H_2_O_2_) decomposition to create new pores on the surface and within the slit pores of the platelets of layered materials. We used a derivative form of hydrotalcite as model compound to demonstrate the method. Hydrotalcite is a member of the family of LDHs, which is a broad family of hydroxides of metals from the groups I-II-III-A, and the transition metals^17^. These compounds are composed of positively charged layers, intercalated with anionic species. The anions may vary from small inorganic ions (CO_3_^2−^ or Cl^−^) to bulky organic ions such as dodecylsulfate (DS)^18^. This family of hydroxides shares the structure of brucite, Mg(OH)_2_. The net charge present in the layers originates from the partial substitution of divalent cations (M^2+^) by trivalent ones (M’^3+^), thereby creating an excess of positive charge. The most common combination of metals is Mg^2+^ and Al^3+^, but many other metals can lead to these layered structures, such as Cr^3+^, Fe^3+^, Ga^3+^, Fe^2+^, Co^2+^, Ni^2+^ or Ca^2+^ and also monovalent cations such as Li^+^. The general formula of these brucite-like compounds is [M_1−x_^2+^M’_x_^3+^(OH)_2_]^x+^A_x/n_^n−^∙mH_2_O where x is the ratio between metal cations (M’^3+^/(M^2+^ + M’^3+^)), n is the charge on the anion A, and m is the number of water molecules located in the interlayer space.

Microwave radiation was used to trigger the decomposition of H_2_O_2_ located in between the layers of the LDH compounds. The decomposition process generates oxygen gas that may force the formation of small holes in the layer structure. This approach was compared with chemically induced decomposition of H_2_O_2_ using iodide ions (I^−^) as catalysts.

## Experimental Section

### Chemicals

The reagents used for the synthesis and modification of hydrotalcite are magnesium nitrate hexahydrate (Mg(NO_3_)_2_∙6H_2_O, 98.5%, Merck), aluminium nitrate nonahydrate (Al(NO_3_)_3_∙9H_2_O, 98.5%, Merck), urea (CH_4_N_2_O, 98%, Sigma-Aldrich), potassium iodide (KI, 99.99%, Sigma-Aldrich), hydriodic acid solution (HI, 57 vol% in H_2_O, Alfa-Aesar), hydrogen peroxide solution (H_2_O_2_, 50 vol%, Sigma-Aldrich) methanol (CH_3_OH, 99.8%, Alfa-Aesar) and demineralized water (DI water). All of them were used without further purification. The microwave-assisted reactions were performed in a Milestone MicroSynth Pro Lab station 230 V/50 Hz with a maximum power of 300 W.

### Synthesis of LDH precursor

The layered double hydroxide of magnesium and aluminium in the carbonate form was prepared based on the method proposed in the literature^19^. The precursors magnesium nitrate hexahydrate, aluminium nitrate nonahydrate and urea were dissolved in DI water. The total concentration of metal ions (Mg^2+^ + Al^3+^) was set to 0.5 M and the concentration of urea to 1 M, the molar ratio of Mg^2+^: Al^3+^ being 4. The precursor solution was then placed in closed quartz vessels for hydrothermal treatment in the microwave (MW) reactor. The temperature was increased at a rate of 5 °C/min to 150 °C and kept at that temperature for 10 minutes. After synthesis, the freshly formed suspensions of LDH were allowed to cool down to room temperature. The powders were collected by centrifugation at 5000 rpm for 10 min and washed with DI water, up to a total of 3 cycles. Afterwards, the samples were dried in a vacuum oven (10^−1^ mbar) at 35 °C for 24 h. This precursor was named LDH-CO_3_.

### Hydrogen peroxide intercalation

A certain quantity of LDH-CO_3_ was thermally treated at 200 °C for 2 h in a vacuum oven (10^−1^ mbar) in order to eliminate all the water present in the interlayer. Subsequently, the dehydrated cold sample was immersed in 50 mL aqueous solution of 50 vol% H_2_O_2_. This suspension was cooled down to 5 °C to avoid premature decomposition of H_2_O_2_ and was stirred overnight to intercalate H_2_O_2_ in the interlayer space. The sample is referred to as LDH-H_2_O_2_.

### Iodide intercalation

The LDH-CO_3_ precursor (1 g) was immersed in 50 mL methanol. Potassium iodide was added to this suspension to obtain a 1 M iodide ion solution; the mixture was vigorously stirred for at least 30 minutes and subsequently heated to 65 °C under reflux conditions and bubbled with N_2_ gas. In parallel, hydriodic acid (2:1 molar ratio with CO_3_^2−^ in the LDH) was mixed with 10 ml methanol and added drop-wise to the hydrotalcite suspension. The reaction was continued for 1 h and the product was collected, centrifuged and washed with methanol 3–5 times until no coloration from hydriodic acid was present. Afterwards, the resulting iodide-intercalated LDH was dried in a vacuum oven overnight at 35 °C. The compound is referred to as LDH-I.

### Synthesis of porous LDH

**Figure 1:**
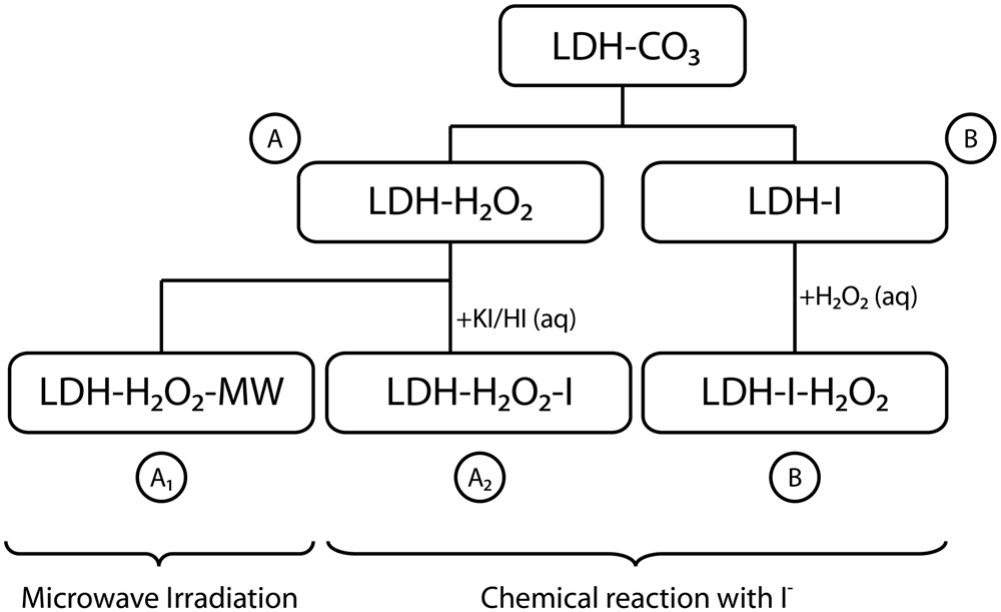
Schematic overview of the three porous LDH synthesis routes.

Three parallel procedures were followed to synthesize porous LDHs by means of H_2_O_2_ decomposition, see Fig. 1. The first route involved microwave-assisted (MW) decomposition of hydrogen peroxide (route A_1_). An aliquot of the LDH-H_2_O_2_ suspension was placed in a round bottom flask inside the MW at 90 °C during 2 h (max. power of 150 W) in order to trigger the decomposition of H_2_O_2_, and to generate oxygen gas. The MW oven was configured with a columnar setup to allow formed oxygen to escape from the system and avoid pressure build-up. The solid was then filtered, washed and subsequently dried under vacuum (10^−1^ mbar) at 35 °C overnight. The final product is referred to as LDH-H_2_O_2_-MW.

A second aliquot was extracted from the original LDH-H_2_O_2_ suspension. The decomposition of H_2_O_2_ was triggered by addition of iodide ions acting as catalyst (route A_2_). The suspension was placed in an Erlenmeyer under mild stirring and 1 ml of 0.001 M KI solution was subsequently added. Immediate bubbling occurred and stirring was continued until all oxygen generation was completed. The powders were filtered, washed and dried under vacuum (10^−1^ mbar) at 35 °C overnight. The final product is referred to as LDH-H_2_O_2_-I.

The third procedure was also based on the chemical reaction between H_2_O_2_ and iodide ions, but the starting compound was LDH-I, the iodide-intercalated form of this layered hydroxide (route B), see Fig. 1. The LDH-I powder was suspended in 50 mL 10 vol% H_2_O_2_ solution under stirring. Stirring was continued until the bubbling stopped. The powders were filtered, washed and dried overnight under vacuum (10^−1^ mbar) at 35 °C. The final product is referred to as LDH-I-H_2_O_2_.

### Product characterization

High Resolution scanning electron microscopy (HRSEM) was performed with a Zeiss MERLIN HR-SEM operating at 1.5 kV. LDH powders were spread over silicon substrates from the suspensions. Thermogravimetric analysis (TGA) was done in Pt cups in a Netzsch STA 449 F3 at a constant heating rate of 5 °C/min in technical air (N_2_/O_2_ = 80/20). Powder X-ray diffraction (XRD) was conducted with a Bruker D2 Phaser (Cu Kα radiation λ  = 0.15405 nm). The patterns were further analysed using the X’Pert Highscore Plus software package. X-ray photoelectron spectroscopy (XPS) was performed with a PHI Quantera Scanning ESCA microprobe with a base pressure below 1 · 10^−8^ mbar. All samples were degassed in the vacuum chamber prior to the measurements. The measurements were done using a monochromatic Al K*α* (1486.6 eV) X-ray source and an EA 125 electron energy analyser. All spectra were acquired in the constant analyser energy (CAE) mode. A CN 10 charge neutralizer system was used to overcome the charging effect in the LDH structures. UV-Vis spectra of samples were recorded with a Cary 50 UV-Vis spectrophotometer in transmission mode. The original suspensions were diluted to obtain an appropriate range of absorbance. Surface area and pore size analysis were performed using the Brunauer-Emmet-Teller (BET) approach. The adsorption/desorption isotherms were collected in a TriStar 3000 analyser at 77 K in N_2_. LDH samples were degassed at 150 °C prior to the measurements.

## Results and Discussion

**Figure 2:**
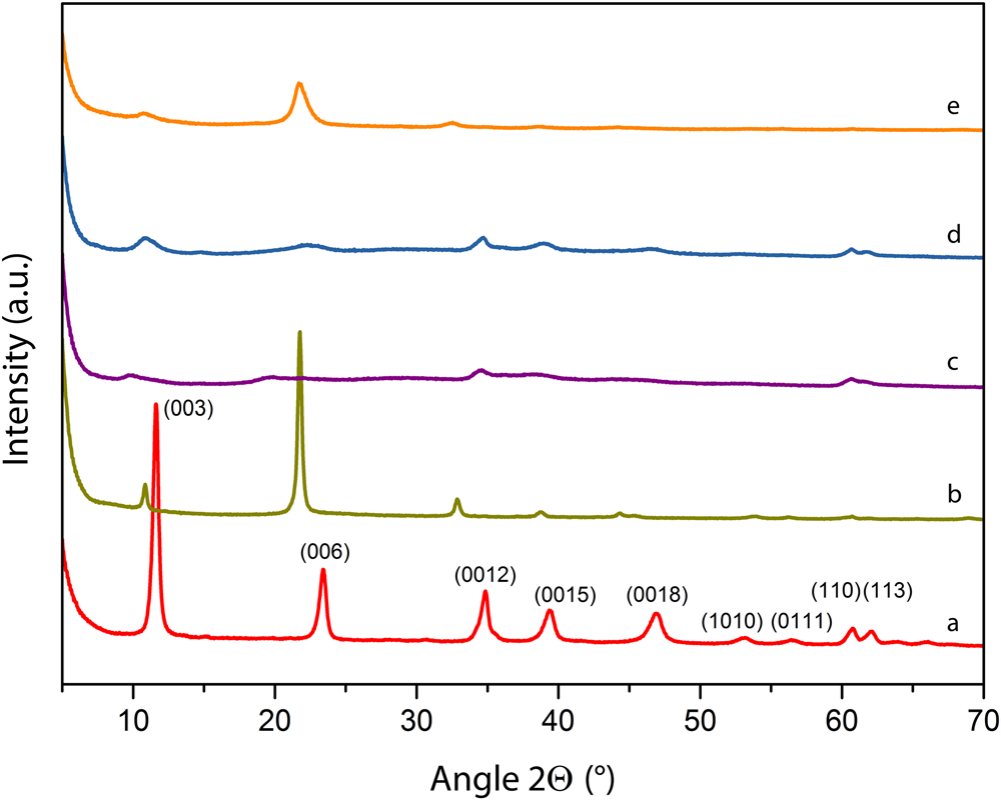
XRD patterns for all LDHs. (**a**) LDH-CO_3_ precursor synthesized by hydrothermal microwave-assisted coprecipitation; (**b**) LDH-I sample prepared from intercalation of iodide ions (I^−^) in between the layers of the LDH-CO_3_ host. The effect of turbostraticity is visible in the peak inversion (**c**) LDH-H_2_O_2_-MW sample synthesized by microwave radiation (route A_1_) of LDH-H_2_O_2_ suspension; (**d**) LDH-H_2_O_2_-I sample prepared by addition of 1 mM KI solution to LDH-H_2_O_2_ suspension (route A_2_) and (**e**) LDH-I-H_2_O_2_ sample prepared by immersion of LDH-I in H_2_O_2_ solution (route B).

Layered double hydroxides were synthesized by coprecipitation from a metal salt solution by means of microwave radiation. The advantage of this technique is the homogeneous ‘molecular’ heating mechanism compared to conventional synthesis in which heat transfer from the outside is dominated by conduction and convection currents. Microwave radiation therefore allows the synthesis of crystalline LDH in relatively short reaction times. Figure 2a shows the XRD pattern of the LDH-CO_3_ precursor, crystalline within 10 min of reaction time. The main Bragg reflections are indexed in the graph, showing a well-crystallized phase characterized by its strong (003) and (006) reflections at 11.6° and 23.4°, respectively. The layers have a *d*-spacing of 0.76(3) nm, which is in accordance with literature data^20^.

Microwave synthesis in closed vessels creates high-temperature and high-pressure conditions. In the coprecipitation process, the hydrothermal conditions reinforce the hydrolysis rate of urea (see eqs 1 and 2), forming high pH nucleation points in solution from which LDH crystal growth can occur.

1 $${\rm{CO}}{({{\rm{NH}}}_{2})}_{2}\mathop{\Longrightarrow }\limits^{{\rm{\Delta }}}{{\rm{NH}}}_{4}{\rm{CNO}}$$

2 $${{\rm{NH}}}_{4}{\rm{CNO}}+2{{\rm{H}}}_{2}{\rm{O}}\to {({{\rm{NH}}}_{4})}_{2}{{\rm{CO}}}_{3}$$

In the hydrolysis of urea, carbonate ions are generated which subsequently take place in the interlayer region and become part of the layer of counter ions between the positively charged hydroxide layers. The hydroxides of magnesium and aluminium crystallize at the new nucleation points. The metal cation ratio (*x*) is generally found to be between 0.2 ≤ *x* ≤ 0.33^20^. The higher the value of *x*, the more excess charge is present in the metal-hydroxide layers, and the smaller the interlayer distance is. When the distance is very small, it is harder to intercalate or functionalize the layers because the attractive electrostatic forces between hydroxide planes and counter-ions are very large. In this research a ratio of *x* = 0.2 (lower limit) was chosen. The overall formula can be expressed as [Mg_0.80_Al_0.20_(OH)_2_](CO_3_)_0.1_∙mH_2_O, with a variable amount of water depending on the synthesis path.

**Figure 3:**
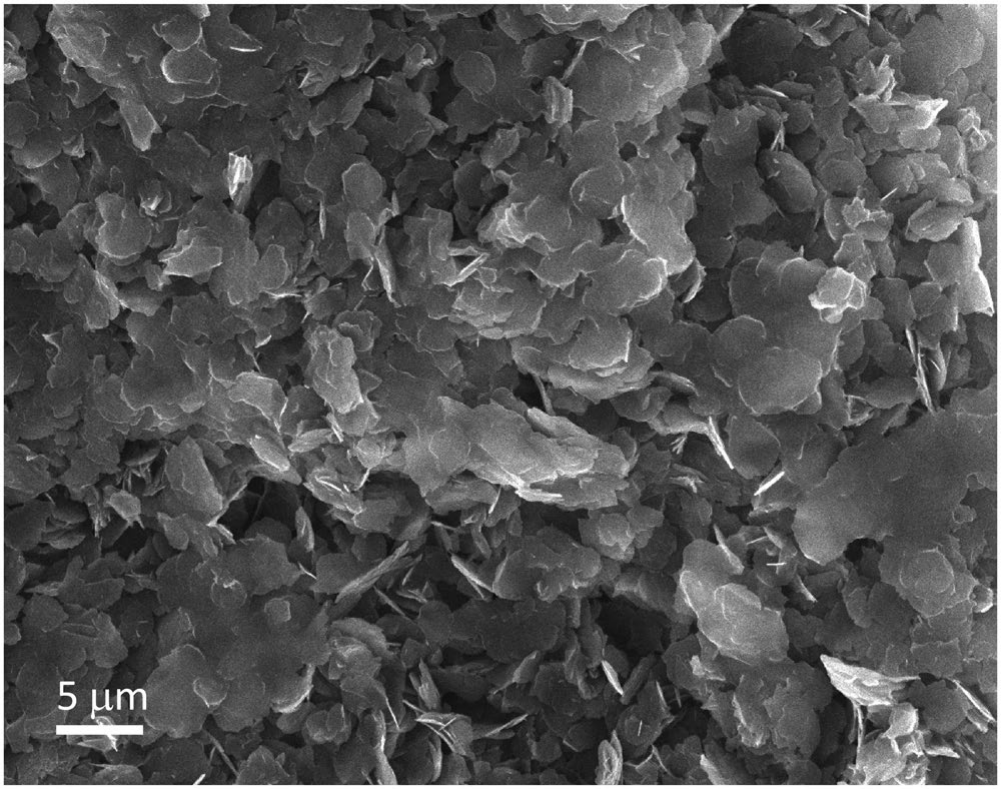
SEM image of LDH-CO_3_ precursor after MW synthesis.

The morphology of the LDH-CO_3_ precursor was examined by HRSEM, and is presented in Fig. 3. Typical LDH platelets can be seen with thicknesses of around 100 nm and round edges. The particle size ranges from 2 to 5 μm, although bigger intergrown platelets of 10 μm and larger can be found at various locations. Dried powders have a heavily agglomerated microstructure with most of the platelets being stacked on top of each other.

Hydrothermal coprecipitation promoted by urea decomposition (eqs 1 and 2) generates LDHs with carbonate ions in the interlayer region. The intercalation of H_2_O_2_ is not impeded by the presence of CO_3_^2−^ ions because of the affinity of H_2_O_2_ to water molecules that are already present in the interlayer region. But in order to intercalate anionic species like iodide ions, decarbonation of the LDH phase is necessary. The precursor LDH-CO_3_ was therefore functionalized via ion exchange. The process was promoted by the presence of hydriodic acid (HI). The protons (H^+^) from HI protonated the carbonate ions, yielding carbonic acid that was eventually liberated in the form of CO_2_ gas from solution. However, at low pH the hydroxide network of LDHs is susceptible to dissolution, generating a morphology change of the particles^21^. To avoid this side-effect, methanol was chosen as solvent instead of water, since alcohol acts as a weaker base for protons^22, 23^. The intercalated iodide entities acted as catalysts for the decomposition of hydrogen peroxide, through the formation of hypoiodite ions that lead to release of oxygen gas and heat^24^. The reactions of this catalytic process are shown in equations 3 and 4:

3 $${{\rm{H}}}_{2}{{\rm{O}}}_{2}({\rm{aq}})+{{\rm{I}}}^{-}({\rm{aq}})\to {{\rm{IO}}}^{-}({\rm{aq}})+{{\rm{H}}}_{2}{\rm{O}}({\rm{l}})$$

4 $${{\rm{H}}}_{2}{{\rm{O}}}_{2}({\rm{aq}})+{{\rm{IO}}}^{-}({\rm{aq}})\to {{\rm{I}}}^{-}({\rm{aq}})+{{\rm{H}}}_{2}{\rm{O}}({\rm{l}})+{{\rm{O}}}_{2}({\rm{g}})\uparrow $$

The change in crystallinity upon iodide intercalation can be monitored by the changes in the XRD spectrum, Fig. 2b. A clear shift of the main reflections to lower angles can be seen, indicating an increase in the interlayer spacing for LDH-I. The (003) peak moves to 10.8° which implies a *d*-spacing of 0.82 nm, providing enough space to accommodate the iodide ions (0.20 nm), while taking into account the layer thickness of 0.48 nm of the metal hydroxides^25^. The intensity of the main reflection (003) was significantly reduced, while the (006) intensity increased slightly. This effect can be due to (1) the large atomic scattering factor of the intercalated iodide ions, which may have added considerably to the reflection intensity by the lighter metals, magnesium and aluminum, in the host layers^26, 27^ and (2) a decrease in the higher-order crystallinity upon ion exchange, which is a phenomenon that has already been observed for I^−^ intercalated LDHs^28^. The structural order of LDHs intercalated with I^−^ is compromised by stacking faults of the cationic metal hydroxide layers and positional disorder of the large iodide anions. This effect is called turbostraticity and it is related to the large size of the anions and the non-directionality of the interaction forces^29^. The necessity of I^−^ excess, choice of solvent and temperature were validated by conducting intercalation experiments in the presence of only HI at room temperature, in the presence of HI and excess KI at room temperature, and using demineralized water instead of methanol as solvent (at 65 °C and with excess KI). These three procedures led to less crystalline materials and are therefore not considered further here. The corresponding x-ray diffractograms are presented in the Supplementary information, Figure S1.

**Figure 4:**
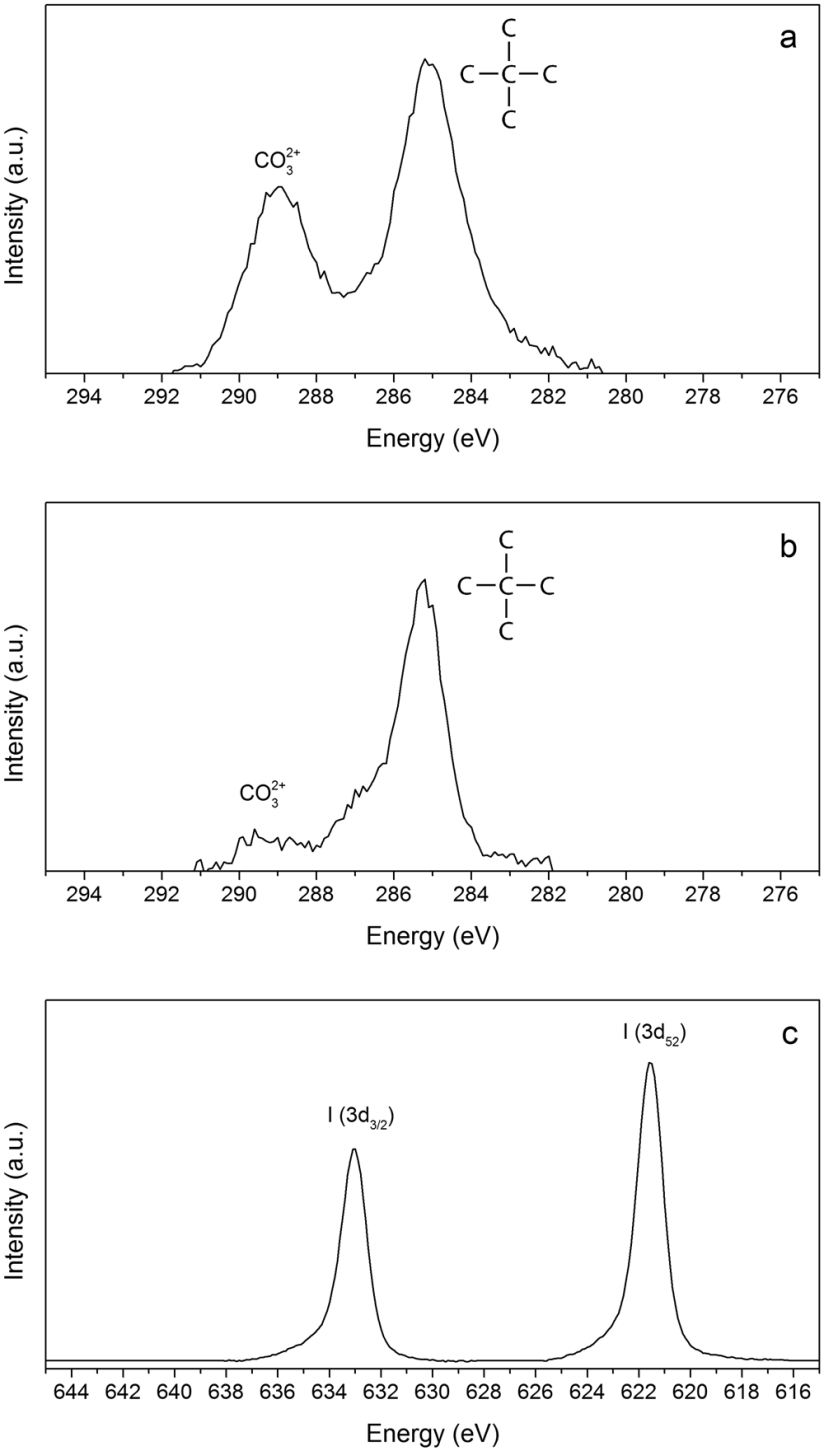
X-Ray Photoemission Spectroscopy for the precursor LDH-CO_3_ and the intercalated form LDH-I (**a**) C 1*s* peaks for LDH-CO_3_ (**b**) C 1*s* peaks for LDH-I and (**c**) I 3*d* peaks for LDH-I.

XPS spectra of LDH-CO_3_ and LDH-I were acquired in order to determine if a quantitative decarbonation of the system had been achieved. Figure 4 shows the binding energy ranges 275–295 eV and 615–645 eV, corresponding to the C 1*s* and I 3*d* orbital energy regions, respectively. Figure 4a,b both display two distinct peaks, belonging to C 1*s* orbitals in different electronic environments. The peaks are attributed to carbon surrounded by carbon atoms at 285 eV, resulting largely from the carbon tape used to attach the LDH powder to the sample holder, and carbonate ions (CO_3_^2−^) at 289 eV. In the case of LDH-I, the carbonate peak had almost completely disappeared, see Fig. 4b. The estimated degree of decarbonation is 80–85%, allowing the presence of iodide ions in the interlayer region. Figure 4c shows the binding energy range between 615 and 645 eV, where two peaks emerged after iodide intercalation. The peaks are assigned to the I 3*d* orbitals; the doublet orbital splitting of 3*d*_3/2_ and 3*d*_5/2_. Iodine found at 621 eV is generally associated with alkali iodides and/or hydrated iodide salts that can be assumed to be reasonably similar to the electronic environment of iodide in the LDH interlayer. Please note that the XPS spectrum before intercalation is not displayed because no peaks were present in the same energy range.

However, the presence of other iodine species cannot be excluded solely by XPS. UV-Vis spectroscopy was performed on the samples to elucidate if the iodide ions had undergone some oxidation to I_2_ in the process. Figure S2 shows that the only absorption peak presented by the intercalated sample suggests the presence of only iodide ions in solution.

**Figure 5:**
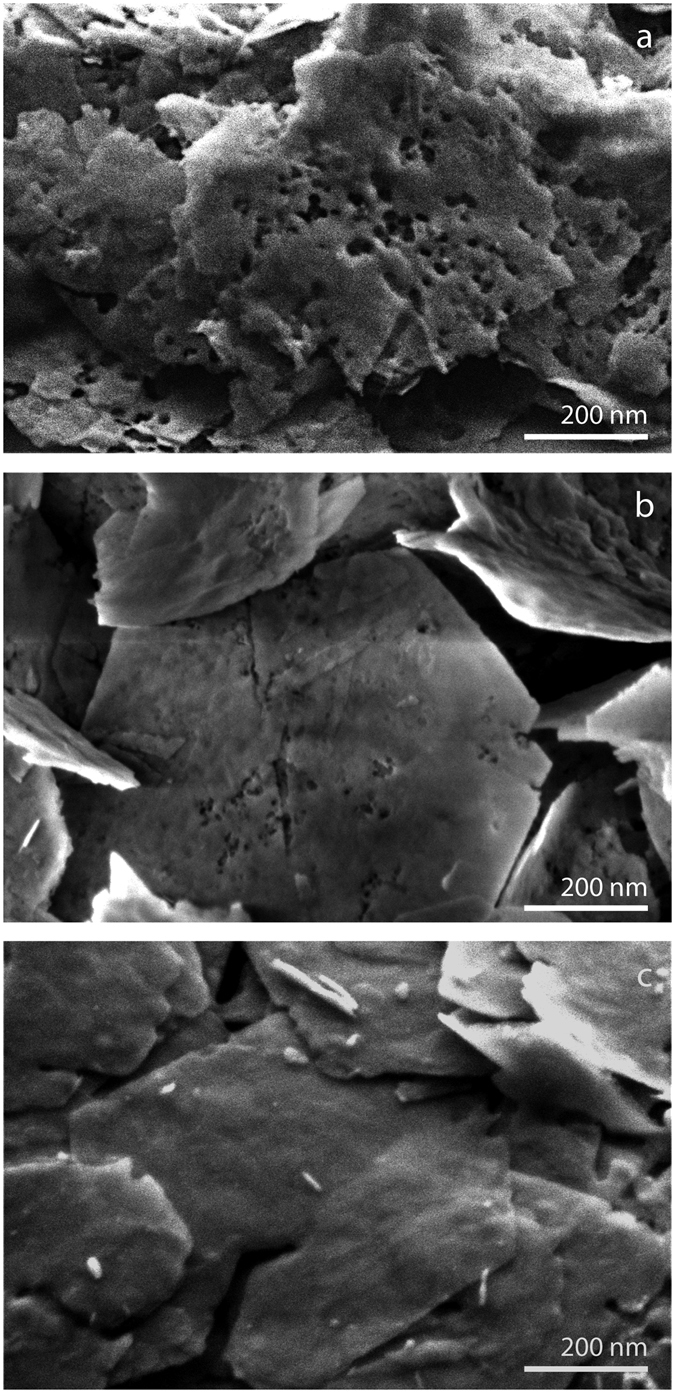
HR-SEM images of (**a**) LDH-H_2_O_2_-MW porous layered material; (**b**) LDH-H_2_O_2_-I porous layered material, and (**c**) LDH-I-H_2_O_2_ porous layered material.

The synthesis of porous LDHs was performed following the methods shown in Fig. 1. Microwave radiation provides homogeneous heating at the molecular level which is expected to trigger uniform decomposition of H_2_O_2_. The microwave radiation method was compared with the decomposition by chemical reaction in order to determine any differences in the pore generation. In the first route (A_1_) the precursor LDH-CO_3_ was dehydrated at 200 °C and in vacuum for 2 h in order to evacuate all water molecules in the interlayer. These conditions were chosen after performing TGA measurements on the precursor powders. These TGA-DTA curves can be found in Figure S3. They show a major weight reduction up to 200 °C that is attributed to water loss from the adsorbed and interlayer water. After dehydration, the powder was suspended in a 50 vol% H_2_O_2_ solution. Dehydration was performed to enhance the diffusion of H_2_O_2_ and water molecules back into the interlayer. Water removal from a layered system has been mentioned as a crucial step to accomplish quantitative intercalation of H_2_O_2_^30^. The LDH-H_2_O_2_ suspension was annealed in the MW reactor for 2 h in an open vessel until all hydrogen peroxide had completely decomposed. The formation of oxygen gas in led to a reduction of the crystallinity, as can be elucidated from the XRD pattern of LDH-H_2_O_2_-MW, see Fig. 2c. The peaks of the main reflections were still present after the treatment and they had shifted to lower angles, indicating that the layered structure was retained and the *d*-spacing was slightly larger with an average of 0.92 nm. The full width at half maximum (FWHM) of the XRD peaks increased, indicating some degree of stacking disorder, most likely due to re-arrangement of the layers resulting from the formation of oxygen gas. The absence of a broad background, typical of amorphous materials, discards the possible dissolution of the LDH and formation of amorphous hydroxides^31^. Figure 5a shows the porous LDH-H_2_O_2_-MW material, with a surface covered by new pores formed by the sudden release of confined oxygen nano-bubbles formed by decomposition of H_2_O_2_ in the interlayer region. The visible pores have a diameter of ~20 nm and can be classified as mesopores. The local build-up of oxygen pressure was high enough to pierce holes in the surface of the layers. Previous studies evaluated the strength of the layers, and found Young’s moduli of 63.4 ± 0.5 GPa for a hydrated system and 139 ± 1 GPa for isolated nanosheets^32^. The overpressure generated by oxygen is enough to overcome the mechanical strength of the nanosheets. Moreover, the build-up of oxygen also contributed to the expansion of the interlayer distance, and the increase of turbostraticity A possible mechanism is that the smallest nanobubbles, with their large internal overpressure, caused the generation of micropores by puncturing the layers, while the larger oxygen bubbles locally expanded the interlayer distances, before the evolved oxygen was released via micropores and edges. The edges of the particles are strongly affected by the treatment and some fragments seem to have been ejected from the structure. In conclusion, the crystallinity and layered structure were retained after the microwave treatment, as evidenced by XRD and HR-SEM. No amorphization of the hydroxide compounds was observed.

The second synthetic route (A_2_) consisted of the addition of a diluted solution of KI to an LDH-H_2_O_2_ suspension. The reaction between iodide ions and H_2_O_2_ is self-propagating due to the fact that heat generated in the reaction enhances the reaction itself. For this reason the concentration of iodide was kept low at [I^−^] = 1 mM. The crystallographic changes were similar to those of LDH-H_2_O_2_-MW. The effect can be seen in Fig. 2d by the similar degree of crystal disorder on the LDH particles, the enlargement of the FWHM and the slight shift of the main reflections to lower angles. The average *d*-spacing that can be calculated from these data is 0.82 nm. Nevertheless, the morphology of the particles showed fewer changes than for LDH-H_2_O_2_-MW, as illustrated in Fig. 5b. The pores seem to be more superficial and occur less frequently in number. In the case of the MW reaction all H_2_O_2_ molecules have the same probability to react since MW radiation interacts with all of them homogeneously. But in the chemical reaction with iodide, the decomposition was less localized between the layers. The presence of H_2_O_2_ not only in the interlayer but also in the bulk of the solution could also be a reason for the formation of more superficial pores on the platelets.

The third synthetic route (B) consisted of the immersion of LDH-I in a 10 vol% H_2_O_2_ solution. The O_2_ generation rate was monitored during the experiment. The O_2_ generation rate gradually reached a maximum of 10 mL/min. This rate increase is an indication of the increase of concentration of free iodide ions in solution with time. A similar but less pronounced loss of structural integrity was observed for catalytic H_2_O_2_ exfoliation of the LDH-I sample where the *d*-spacing remained constant while the FWHM of the main reflections increased, see Fig. 2e. The turbostratic effect was conserved, meaning that the natural crystallographic disorder of the platelets was added to the perturbations in the structure coming from the chemical reaction between H_2_O_2_ and iodide. The morphology of the platelets shown in Fig. 5c suggests that the absence of intraplatelet holes is due to the diffusion of iodide ions within the layers, creating further crystallographic disorder and reacting outside the platelets without producing any pores.

The increase of porosity in these layered systems was analysed using N_2_ BET analysis. Absorption-desorption isotherms showed that all LDHs exhibit a type IV isotherm and H3 hysteresis loop. This is a common signature for plate-like particles and with slit-shaped pores, according to the IUPAC classification^33^ (see example in Figure S4). The surface areas of all compounds are summarized in Table 1. The contributions of the micropores and meso-/macropores to the total surface area are listed. As expected, the precursor LDH-CO_3_ has the lowest surface area. The meso/macroporous regime (pore size >2 nm) is dominant and is attributed primarily to the external surface area of the platelets. The micropores (pore size <2 nm) are attributed to the near edge zone of the interlayer region.

**Table 1:** Summary of surface properties and micropore volume of all LDHs.

LDH	Surface area (m^2^ g^−1^)	Micropore volume (cm^3^ g^−1^)	Micropore area (m^2^ g^−1^)	Meso/macropore area (m^2^ g^−1^)	Contribution total surface area (%)	
					Micropores	Meso/Macropores
LDH-CO_3_	9.0	0.000294	0.3	8.7	4	96
LDH-I	11.1	0.000333	1.6	9.5	14	86
LDH-I-H_2_O_2_	37.1	0.00198	3.3	33.9	9	91
LDH-H_2_O_2_-KI	56.6	0.0168	39.0	17.6	69	31
LDH-H_2_O_2_-MW	66.5	0.0177	37.4	29.1	56	44

On the one hand, the functionalization of layered LDH-CO_3_ with iodide does not bring important changes to the surface area or the pore size distribution. The turbostraticity of the platelets may aid in exposing part of the microporous openings of the interlayer region. On the other hand, all three treatments with H_2_O_2_ led to a notable increase of the final specific surface area. LDH-I-H_2_O_2_ shows a relative increase of specific surface area by 300%, mostly due to the increase of meso/macroporous surface area. This cannot be attributed to the formation of new pores as evidenced in Fig. 5c, but to (local) interlayer distance enlargement and turbostratic rearrangement of nanosheets that form the platelets. The mismatch between the nanosheets induced by these perturbations led to exposure of new surface areas on the edges of the platelets. The change in crystallinity as shown in Fig. 2e supports this interpretation. Treatment of LDH-H_2_O_2_ with iodide ions increased the surface area of LDH by 500%. The clear increase of the micropore surface area can be attributed to two factors. Firstly, the generation of micropores on the surface of the platelets was caused by O_2_ nano-bubbles around the edges and on the surface of the platelets. And secondly, these new pores allowed exposure of the interlayer region between nanosheets that was previously not accessible to N_2_. Finally, the microstructure most affected by these oxygen nano-bubbles is the MW treated LDH-H_2_O_2_. The final surface area increased by 640% to 66.5 m^2^ g^−1^, with a larger contribution of the micropore surface area to the final total surface area. It is remarkable that the contribution of meso/macropores in this compound is higher than for LDH-H_2_O_2_-KI. The more homogeneous and higher reactivity of H_2_O_2_ enhanced by MW led to more rapid release of confined oxygen between the platelets. The more violent pressure release formed larger pores in the surface of the platelets and exposed the inner interlayer region of the platelets. This effect can also be seen by the rise in micropore volume, see Table 1. For both LDH-H_2_O_2_ intercalated compounds, the final micropore volume had increased by a factor of 50 after H_2_O_2_ decomposition. This is associated with the swelling and mismatch of the nanosheets after treatment, and aided by the accessibility of the interlayer intraplatelets, thanks to the presence of pores.

## Conclusions

**Figure 6:**
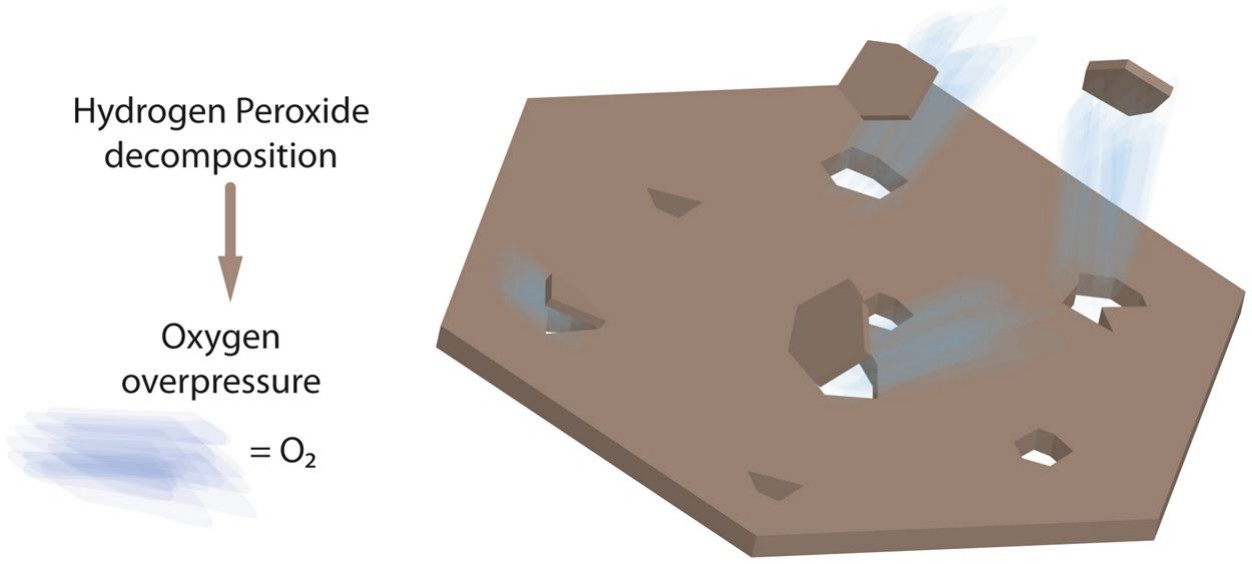
Schematic representation of the micropore formation process in Mg-Al LDH as proposed in this report.

We report a rapid and environmentally friendly method for the synthesis of porous Mg-Al LDHs based on the decomposition of hydrogen peroxide into gaseous oxygen triggered by microwave radiation, as schematically sketched in Fig. 6.

The crystallinity of the Mg-Al LDHs was hardly affected by the pore generation process. Only some stacking disorder of the nanosheets was observed. The integrity of the remaining layered structure was not affected by the change in morphology and the layered compound can potentially be functionalized further after the creation of pores. The most effective way to synthesize porous LDHs is by means of microwave-enhanced hydrogen peroxide decomposition. The final surface area increased up to 640% and the micropore volume increased by a factor of 50. The decomposition of H_2_O_2_ in the interlayer region through MW radiation appears to be faster than decomposition triggered by chemical reaction, giving rise to larger pores crossing the platelets. This technique could potentially be applied to increase the surface area of other layered materials composed of elements that are stable against oxidation and that allow intercalation of hydrogen peroxide, and should be tested also for other LDH compositions in order to determine its window of applicability.

## Electronic supplementary material

Supporting information file
