# Supplementary material for: Porous Layered Double Hydroxides Synthesized using Oxygen Generated by Decomposition of Hydrogen Peroxide
Source: Sci Rep. 2017 Mar 28;7:481. doi: 10.1038/s41598-017-00283-9 (PMC5428037; doi:10.1038/s41598-017-00283-9)
Supplement: Supplementary file 1 — Supporting information file [file 41598_2017_283_MOESM1_ESM.pdf]

**SUPPORTING INFORMATION**

# Porous Layered Double Hydroxides Synthesized Using Oxygen Generated by Decomposition of Hydrogen Peroxide

P. Gonzalez-Rodriguez,[1-2] M. de Ruiter,[2] T. Wijnands,[2] J.E. ten Elshof [2]

[1] Materials innovation institute (M2i). Elektronicaweg 25, 2628 XG Delft, the Netherlands.

[2] Inorganic Materials Science Group, MESA+ Institute for Nanotechnology, University of Twente, P.O. Box 217, 7500 AE Enschede, The Netherlands.

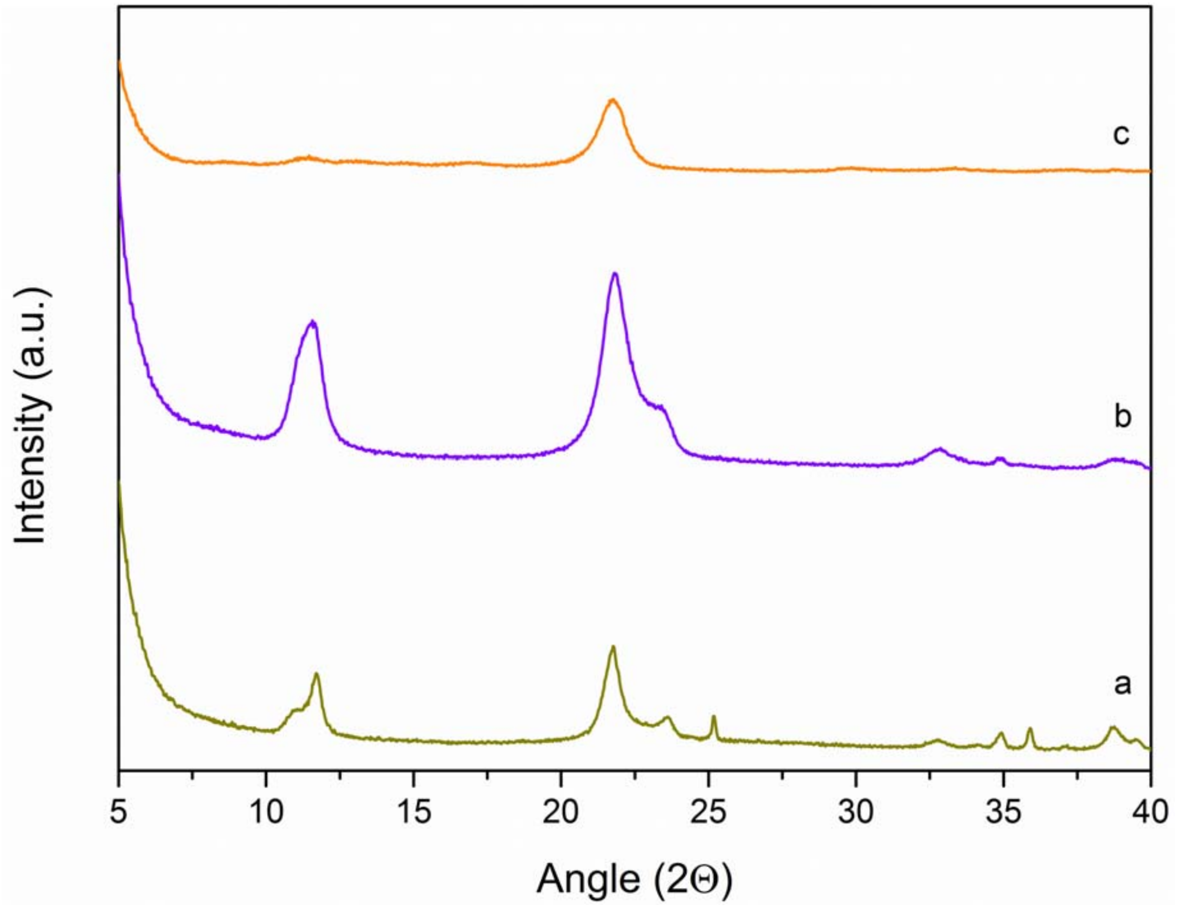


**Figure S1. XRD patterns for LDH-I compounds synthetized by other methods than proposed in the core of the research. (a) LDH-I synthesized in methanol at room temperature and with a stoichiometric amount of HI (to $CO_3^{2-}$ ions) with same intercalation time, 1 h. The intercalation seems incomplete with side peaks caused by side products; (b) LDH-I synthesized in methanol at room temperature and with a stoichiometric amount of HI (to $CO_3^{2-}$ ions) and excess of KI salt, with same intercalation time, 1 h. Lower crystallinity than for synthesis at 65°C; (c) LDH-I synthesized in water at 65°C, stoichiometric amount of HI (to $CO_3^{2-}$ ions) and excess of KI salt with the same intercalation time, 1 h. Low pH in water seems to affect the overall structure of the LDH.**

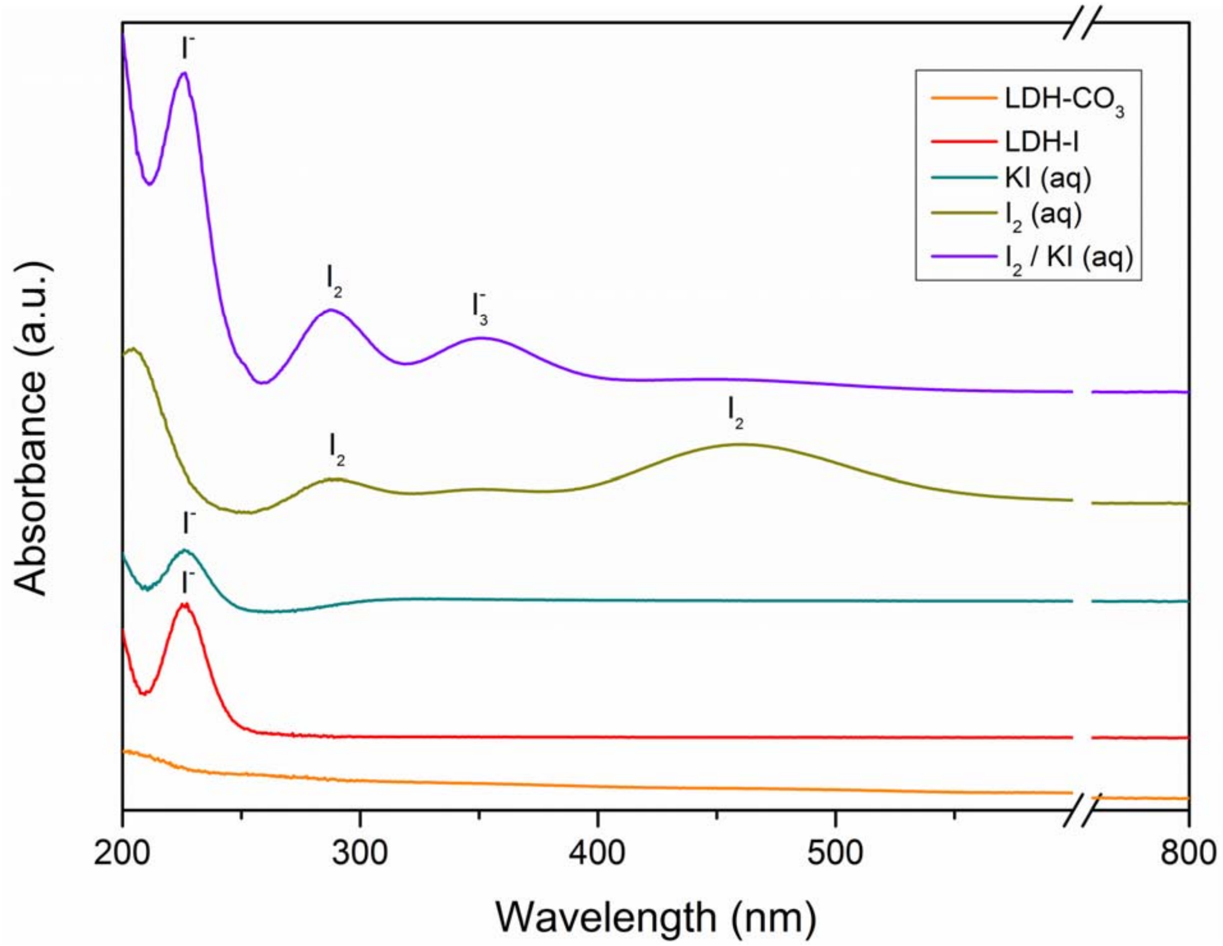


**Figure S2. UV-Visible spectra for the LDH-CO3 (bottom) and LDH-I functionalized sample (second from bottom). For comparison purposes, the UV-Vis spectra of diluted solutions/suspensions of Iodide ($I^-$), Iodine ($I_2$) and tri-iodide ions ($I_3^-$) with the purpose to show that no other side products are generated in the intercalation process.**

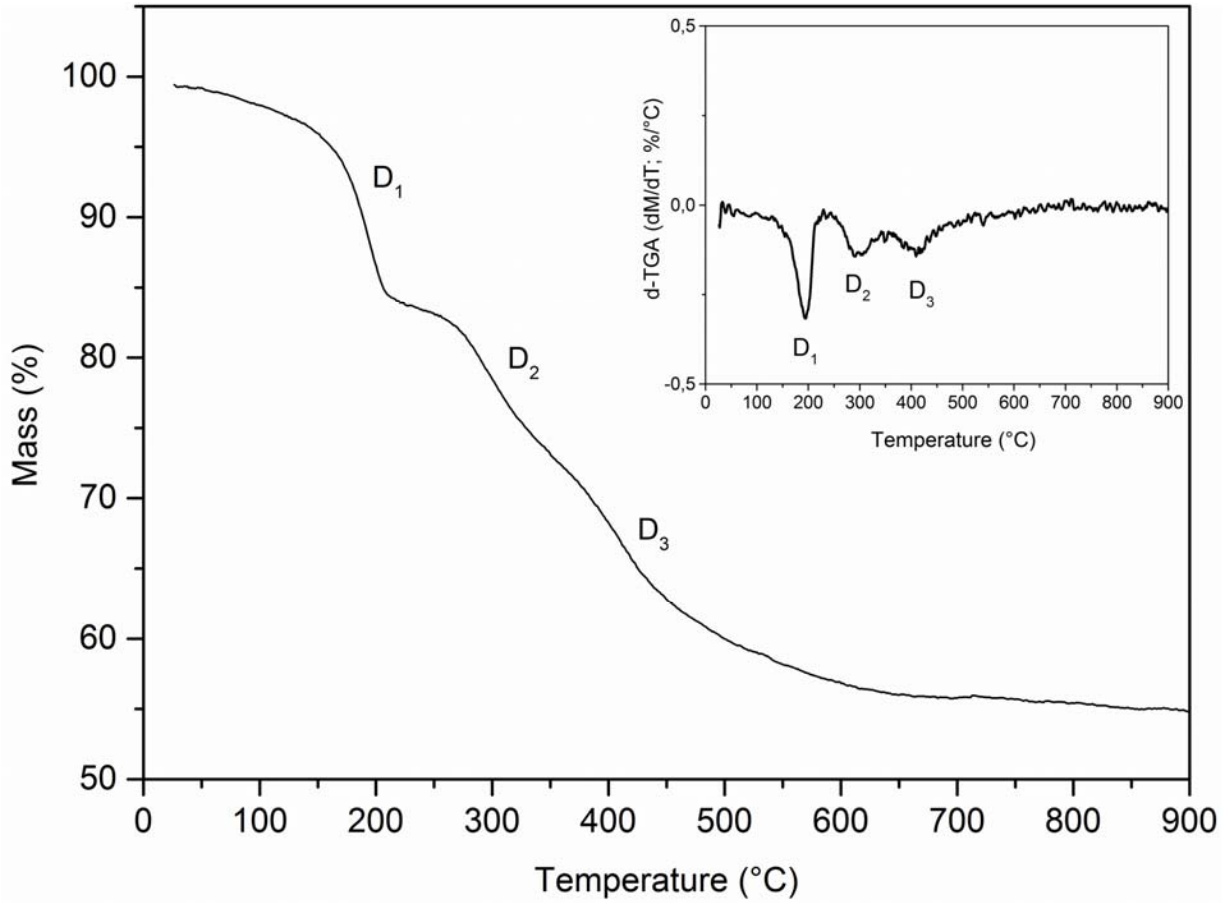


**Figure S3. Thermogravimetric Analysis (TGA) and Differential-TGA (in inset) curves of LDH-$CO_3$. Dehydration of LDH starts with water removal from the surface of the platelets and ends with the removal of interlayer water (crystalline water) at 200°C (peak D1). Peaks $D_2$ and $D_3$ correspond to the dehydroxylation of the network and subsequent thermal decarbonation, respectively.**

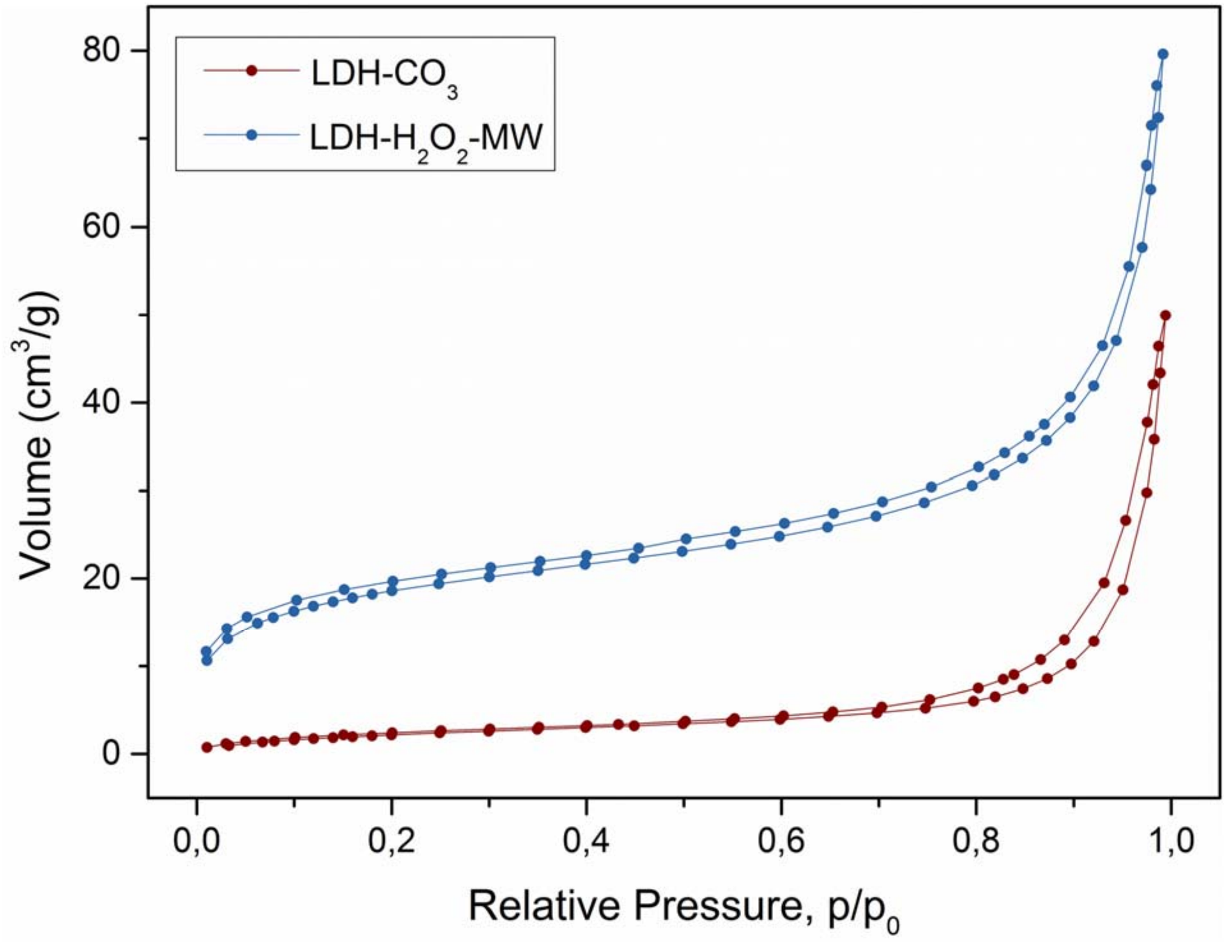


**Figure S4. $N_2$ Adsorption-desorption isotherms for LDH-$CO_3$ and LDH-$H_2O_2$-MW.**
